# Alteration of Gut Microbiota and Inflammatory Cytokine/Chemokine Profiles in 5-Fluorouracil Induced Intestinal Mucositis

**DOI:** 10.3389/fcimb.2017.00455

**Published:** 2017-10-26

**Authors:** Hong-Li Li, Lan Lu, Xiao-Shuang Wang, Li-Yue Qin, Ping Wang, Shui-Ping Qiu, Hui Wu, Fei Huang, Bei-Bei Zhang, Hai-Lian Shi, Xiao-Jun Wu

**Affiliations:** Shanghai Key Laboratory of Compound Chinese Medicines, The Ministry of Education (MOE) Key Laboratory for Standardization of Chinese Medicines, Institute of Chinese Materia Medica, Shanghai University of Traditional Chinese Medicine, Shanghai, China

**Keywords:** gut microbiota, 5-fluorouracil, intestinal mucositis, inflammatory chemokines/cytokines, fecal transplantation

## Abstract

Disturbed homeostasis of gut microbiota has been suggested to be closely associated with 5-fluorouracil (5-Fu) induced mucositis. However, current knowledge of the overall profiles of 5-Fu-disturbed gut microbiota is limited, and so far there is no direct convincing evidence proving the causality between 5-Fu-disturbed microbiota and colonic mucositis. In mice, in agreement with previous reports, 5-Fu resulted in severe colonic mucositis indicated by weight loss, diarrhea, bloody stool, shortened colon, and infiltration of inflammatory cells. It significantly changed the profiles of inflammatory cytokines/chemokines in serum and colon. Adhesion molecules such as vascular cell adhesion molecule-1 (VCAM-1), intercellular adhesion molecule-1 (ICAM-1), and VE-Cadherin were increased. While tight junction protein occludin was reduced, however, zonula occludens-1 (ZO-1) and junctional adhesion molecule-A (JAM-A) were increased in colonic tissues of 5-Fu treated mice. Meanwhile, inflammation related signaling pathways including NF-κB and mitogen activated protein kinase (MAPKs) in the colon were activated. Further study disclosed that 5-Fu diminished bacterial community richness and diversity, leading to the relative lower abundance of Firmicutes and decreased Firmicutes/Bacteroidetes (F/B) ratio in feces and cecum contents. 5-Fu also reduced the proportion of Proteobacteria, Tenericutes, Cyanobacteria, and Candidate division TM7, but increased that of Verrucomicrobia and Actinobacteria in feces and/or cecum contents. The fecal transplant from healthy mice prevented body weight loss and colon shortening of 5-Fu treated mice. In addition, the fecal transplant from 5-Fu treated mice reduced body weight and colon length of vancomycin-pretreated mice. Taken together, our study demonstrated that gut microbiota was actively involved in the pathological process of 5-Fu induced intestinal mucositis, suggesting potential attenuation of 5-Fu induced intestinal mucositis by manipulating gut microbiota homeostasis.

## Introduction

Gastrointestinal microbiota plays an important role in the maintenance of human health (Figlewicz, 2008; Zhao, 2013; Patel et al., 2016). Healthy gastrointestinal microbiota characterized by high rich and diverse bacteria (Vandeputte et al., 2016) interacts with mucosal epithelium and is responsible for normal substance metabolism, immune response and intestinal angiogenesis (Stringer et al., 2009a,b; Candela et al., 2014). Disturbed gut microbiota has been revealed to induce many disorders, such as metabolic diseases (obesity and diabetes) (Philippot et al., 2013), inflammatory bowel diseases (Tung et al., 2011), multiple sclerosis, and even psychiactric diseases such as depression (Wang and Kasper, 2014). More and more evidences suggest that sustained homeostasis of gut microbiota seems to benefit the recovery of many diseases.

Cancer chemotherapeutic agents have been found to interfere with the homeostasis of gut microbiota. For instance, irinotecan, a cytotoxic chemotherapy agent for colon cancer, can induce the alteration of β-glucuronidase producing bacteria of intestinal microflora (Stringer et al., 2007). Ipilimumab, a CTLA-4 blocker, even has to exert its anticancer effect through the interaction between *Bacteroides fragilis* (*B. fragilis*) and *B.fragilis*-specific T cells (Vétizou et al., 2015). 5-fluorouracil (5-Fu), the first-line chemotherapeutic agent for the therapy of metastatic colorectal cancer, induces gastrointestinal adverse events such as diarrhea, hemorrhage and intestinal mucositis in clinic, which not only diminish its therapeutic efficacy but also increase patient's suffering (Sonis et al., 2004; Stringer et al., 2009b). Administration of probiotics ameliorates 5-Fu induced intestinal mucositis in mice (Justino et al., 2014; Yeung et al., 2015), suggesting possible causality between the gastrointestinal microbiota and the disease. In rats, 5-Fu treatment changes the relative abundance of microbiota from several genera in gastrointestine, including *Clostridium, Lactobacillus, Enterococcus, Bacteroides, Straphylococcus, Streptococcus*, and *Escherichia* (Stringer et al., 2007). However, due to the limited techniques at that time, the profiling of the gastrointestinal microbiota was incomplete. In addition, the detailed function of gut microbiota in 5-Fu-induced gastrointestinal mucositis has not been well clarified yet.

In 5-Fu-induced mucositis rodents, chemokines/cytokines such as chemokine-1, 2, 9 (CXCL1, CXCL2, CXCL9), and interleukine-4 (IL-4) are elevated, which is accompanied with intestinal epithelium damage. Further study disclosed that CXCL9 is closely related to the intestinal damage, while IL-4 as a pro-inflammatory cytokine can increase intestinal epithelium permeability (Prisciandaro et al., 2012; Soares et al., 2013; Wang and Kasper, 2014; Lu et al., 2015; Sakai et al., 2016). NF-κB and mitogen activated protein kinase (MAPK) pathways can be activated in the small intestine of 5-Fu induced mucositis (Liu et al., 2013). However, the reciprocal association among the overall profiles of 5-Fu-induced inflammatory cytokines/chemokines, alteration of tight junction and adhesion proteins and cellular signaling pathways has not been elucidated, especially in colon tissue.

Although disturbed homeostasis of gut microbiota has been suggested to be closely associated with the adverse effect of 5-Fu, current knowledge of the overall profiles of 5-Fu-disturbed gut microbiota is limited, and so far there is no direct convincing evidence that can prove the causality between 5-Fu-disturbed microbiota and colonic mucositis. The present study was aimed to provide the overall profile of 5-Fu-disturbed gut microbiota by direct sequencing of 16S rRNA gene in cecum contents and feces of colonic mucositis mice using high throughput Miseq sequencing technologies. Meanwhile, the influence of 5-Fu on the inflammatory cytokines/chemokines, adhesion molecules, tight junction molecules as well as MAPK and NF-κB pathways in colonic tissues of mice was investigated. And the fecal transplantation experiments were conducted to elucidate the causality between gut microbiota and colonic mucositis. Our findings confirmed the important role of gut microbiota in 5-Fu induced intestinal mucositis and may provide novel therapy regimen for patients suffered from 5-Fu induced intestinal mucositis.

## Materials and methods

### Animals and mucositis induction

Male BALB/c mice, 4-week old, obtained from Shanghai SLAC Laboratory Animal Co. Ltd. (SYXK2014-008, Shanghai, China) were housed under a 12 h light/dark cycle at room temperature (23 ± 2°C) with access to food and water *ad libitum*. Two weeks later, the mice were randomly divided into two groups, namely control group and 5-Fu group (*n* = 10/group). According to Huang et al's method (Huang et al., 2009), to induce mucositis, the 5-Fu group mice were intraperitoneally administered with 5-Fu (50 mg/kg) once daily for 3 days. Meanwhile, the control group mice were intraperitoneally administered with 0.9% saline. All animal experiments were conducted complying with the Institutional Animal Care guidelines approved by the Experimental Animal Ethical Committee of Shanghai University of Traditional Chinese Medicine.

### Mucositis assessment and samples collection

Body weight, diarrhea and bloody stool of mice were recorded daily for the assessment of mucositis. Diarrhea grade was evaluated based on the consistency of stool, using the modified parameters as described previously (Leocádio et al., 2015): 0, normal; 1, slightly wet; 2, moderate wet; 3, loose; 4, watery stool. At the last day (day 7), the grade of blood stool was assessed by a commercial testing paper (BASO diagnostics Inc. China) with the following scores: 0, normal; 1, slight bleeding; 2, moderate bleeding; 3, severe bleeding; 4, visible bleeding. Meanwhile, the feces were collected and stored at −80°C. Then the mice were sacrificed under anesthesia, and the entire small intestine and colon were excised after removal of fat tissue and their length were measured. The colon tissues near the cecum were either fixed in 10% formalin (w/v) or snap frozen in liquid nitrogen for further analysis.

### Protein chip analysis

Colon tissues were homogenized with cell lysis buffer containing protease inhibitor cocktail on ice and centrifuged at 12,000 rpm for 15 min at 4°C. The supernatant was collected and subjected to concentration measurement using BCA method. Afterwards, all protein samples were diluted to the same concentration. Inflammatory/anti-inflammatory cytokines in the samples were measured by RayBio® Mouse Cytokine Antibody Arrays according to the manufacturer's protocol.

### Histopathological assessment

Fixed colon tissue samples were embedded in paraffin, sectioned in 4 μm-thick slices, and stained with hematoxylin-eosin. The morphological alteration and inflammatory cell infiltration were observed under microscope (Olympus BX61VS).

### Immunohistochemistry

The endogenous peroxidases in 4 μm-thick slices were deactivated by incubation with 3% H_2_O_2_ for 10 min. For antigen retrieval, the sections were soaked in 10 mM citrate buffer solution (pH 6.0) and heated twice in a microwave oven. After washed thoroughly with PBS (pH7.4), the sections were blocked with 3% BSA in tris buffered saline (TBS) for 20 min, then incubated with anti-myeloperoxidase antibody (anti-MPO) (1:200, #SH0022, Skyhobio) and anti-p65 (1:400, #SH0023, Skyhobio) antibodies overnight at 4°C followed by incubation with HRP-conjugated secondary antibody (#K5007, Dako) for 50 min. The sections were further incubated with DAB-H_2_O_2_ solution (#K5007, Dako), counterstained with hematoxylin, dehydrated with ethanol and sealed in resinene for microscopic observation.

### Quantitative polymerase chain reaction (qPCR)

Total RNA was extracted from colon tissues using TRIzol reagent (Life Technologies). cDNA was generated from total RNA with the RevertAid First Strand cDNA Synthesis Kit (Thermo). The primers (GeneRay) used in PCR amplification were listed in Table 1. Quantitative PCR was performed with SYBR Premix EX Taq under the following conditions: 95°C, 30 s; then followed by 40 cycles (95°C, 5 s; 60°C, 34 s); finally 95°C, 15 s; 60°C, 1 min; 95°C, 15 s. Quantity of target genes calculated by the comparative C_t_ method was normalized to that of β-actin (internal reference) in the same sample (Araújo et al., 2015).

**Table 1:** The primers used in qPCR analysis.

**Genes**	**Forward primer**	**Reverse primer**
IFN-γ	ATTGCGGGGTTGTATCTGGG	GGGTCACTGCAGCTCTGAAT
IL-1β	TTTGAAGTTGACGGACCCC	TGTGCTGCTGCGAGATTTG
IL-6	ACCACGGCCTTCCCTACTTC	CATTTCCACGATTTCCCAGA
TNF-α	GGAACACGTCGTGGGATAATG	GGCAGACTTTGGATGCTTGTT
CXCL5	GTTCATCTCGCCATTCATGC	GCGGCTATGACTGAGGAAGG
CXCL9	GGAGTTCGAGGAACCCTAGTG	GGGATTTGTAGTGGATCGTGC
CXCL13	GGCCACGGTATTCTGGAAGC	GGGCGTAACTTGAATCCGATCTA
CXCL1	GGCTTCCTTATGTTCAAACAGGG	GCCGTTACTCGGGTAAATTACA
CD11b	GGTCGGCAAGCAACTGATTT	CAACTTGCATTATGGCATCCA
IL-22 R1	GACTCCATTTGCGTCATCGC	CCTGTCACCGTGTGTCATCA
IL-10 R2	TGGAGCCGTGGACAACTTAC	ATGGCCACAATCCAGGAAGG
VCAM-1	GAACCCAAACAGAGGCAGAG	GGTATCCCATCACTTGAGCAG
ICAM-1	CGCTGTGCTTTGAGAACTGT	AGGTCCTTGCCTACTTGCTG
VE-Cadherin	CGCCAACATCACGGTCAA	ACGGTTAGCGTGCTGGTTC
Occludin	ATGGCAAGCGATCATACCC	TTCCTGCTTTCCCCTTCG
β-actin	CGGTTCCGATGCCCTGAGGCTCTT	CGTCACACTTCATGATGGAATTGA

### Multiplex immunoassays

Serum was collected by centrifugation at 4,000 rpm for 10 min at 4°C. Colon segments were homogenized in cell lysis buffer, then the supernatants were collected through centrifugation at 12,000 rpm for 15 min. Concentrations of 13 cytokines in the supernatants and serum were measured by ProcartaPlex® Mix&Match Mouse 13-plex [including interleukin-6 (IL-6), tumor necrosis factor-α (TNF-α), interleukin-10 (IL-10), interleukin-12p-70 (IL-12p70), interleukin-21 (IL-21), interleukin-22 (IL-22), interleukin-31 (IL-31), granulocyte colony stimulating factor (G-CSF), granulocyte-macrophage colony stimulating factor (GM-CSF), Leptin, RANTES, chemokine-5 (CXCL5), and chemokine-1 (CXCL1)] according to the manufacturer's recommendation.

### ELISA assay

Concentration of CXCL9 in serum and supernatants of colonic tissues was quantified by CXCL9 ELISA assay kit (Abcam, UK) according to manufacturer's instruction.

### Western blot analysis

Colon tissues were homogenized and lysed in RIPA buffer supplemented with protease inhibitor cocktail on ice. After centrifugation at 12,000 rpm for 15 min at 4°C, the supernatant was collected and its protein concentration was determined by BCA method. Total protein (60 μg) from each sample was separated by SDS-PAGE and transferred onto PVDF membrane by wet transfer approach. Then PVDF membranes were blocked with 5% (w/v) bovine serum albumin (BSA) solution and incubated with different primary antibodies against p-p65 (1:1000, #3033L, Cell Signal Technology), p-IκBα (1:500, #2859S, CST), p-p38 MAPK (1:1000, #4511, CST), phosphorylated extracellular signal-regulated kinase (p-ERK1/2, 1:1000, #9154, CST), phosphorylated jun N-terminal kinase (p-JNK, 1:1000, #4668, CST), p38 MAPK (1:1000, #9212, CST), extracellular signal-regulated kinase (ERK1/2, 1:1000, #4695, CST), stress activated protein kinase/jun N-terminal kinase (SAPK/JNK, 1:1000, #9252S, CST), inducible NO synthase (iNOS, 1:1000, #ab204017, Abcam), VCAM-1(1:2000, #3540-1, Epitomics), ICAM-1 (1:1000, #3482-1, Epitomics), Occludin (1:2000, #GTX85016, GeneTex), ZO-1 (1:500, #ab59720, Abcam), JAM-A (1:500, # sc-37049, Santa cruz) and β-actin (1:2000, #12413, CST) overnight at 4°C. After washed with 1 × PBS containing 0.1% (v/v) Tween-20, the membranes were incubated with respective secondary antibodies. The protein bands were visualized with ECL-prime kit. Quantification of target protein was performed by measuring integral optic density of respective target proteins with Tanon Gis software.

### 16S rRNA Miseq sequencing and bioinformatic analysis

Microbial genomic DNA was extracted from cecum contents and feces using a QIAamp DNA Stool Mini Kit according to the manufacturer's instructions. The resultant DNA extracts were used for the PCR amplification. Quantification of the PCR products was performed on FTC-3000TM real-time PCR instrument. The V3-V4 region of 16S rRNA gene of gut microbiota was sequenced using Illumina MiSeq 2 × 300 bp high throughput platform. The bioinformatic analysis was conducted as described previously (MacIntyre et al., 2015). The generated 16S rRNA gene sequences were analyzed using the bioinformatic software package Mothur with MiSeq SOP Pipeline. The paired reads were assembled using make.contigs. Screen.seqs command was used to remove low quality reads using the following filtering parameters, maxambig = 0, minlength = 200 and maxlength = 580, maxhomop = 8. The remained sequences were simplified using the unique.seqs command to generate a unique set of sequences, then aligned with the SILVA databases (version 119). The screen.seqs command was implemented again to keep within our defined criteria using the following parameters: start = 12,878, end = 28,464. The filter.seqs was used to remove empty columns from our alignment. Further de-noise sequences were pre-clustered using the pre.cluster command (http://www.mothur.org/wiki/Pre.cluster) allowing for up to 4 differences between sequences. Then reads were checked for chimeras using UCHIME algorithm and the chimeric sequences were removed by the chimera.uchime command with default parameters. To classify (classify.seqs) the sequences, the SILVA 119 database was used with a confidence threshold of 80%. The non-bacterial sequences were deleted. The distance matrix between the aligned sequences was generated by the dist.seqs command. Finally, these sequences were clustered to OTUs (operational taxonomic units) at 97% sequence identity (furthest neighbor method). A majority of consensus taxonomy for each OTU was obtained by the classify.otu command with default parameters.

### Fecal transplantation

For healthy fecal transplantation experiment, 24 mice were randomly divided into three groups: Control, 5-Fu and 5-Fu+feces (*n* = 8/group). Both 5-Fu group and 5-Fu+feces group mice were injected intraperitoneally with 5-Fu (50 mg/kg/day) for 3 days. For 5-Fu+feces group mice, they were additionally administered with the fecal suspension from normal mice via oral gavage from day 1 to day 7 once a day. For 5-Fu-treated fecal transplantation experiment, 40 mice were randomly divided into four groups, namely Control, 5-Fu, Con-feces and 5-Fu-feces (*n* = 10/group). The mice in Control and 5-Fu groups were treated as aforementioned. Fecal pellets from Control group and 5-Fu group mice were collected and suspended in sterile PBS. For Con-feces group and 5-Fu-feces group mice, they were pretreated with vancomycin (100 mg/kg) for 3 days (Ubeda et al., 2013; Warn et al., 2016), then were administered with respective fecal suspension from Control group or 5-Fu group mice by oral gavage for 11 days. Body weight, diarrhea, and bloody stool of mice were recorded daily. At last, the mice were sacrificed under anesthesia and the length of entire colon after removal of fat tissue was measured.

### Statistical analysis

Each value was presented as mean ± S.E.M. Differences between two groups were analyzed by un-paired Student's *t*-test using PrismDemo 5. In all cases, the value of *P* < 0.05 was considered statistically significant.

## Results

### 5-Fu induced colonic mucositis

**Figure 1:**
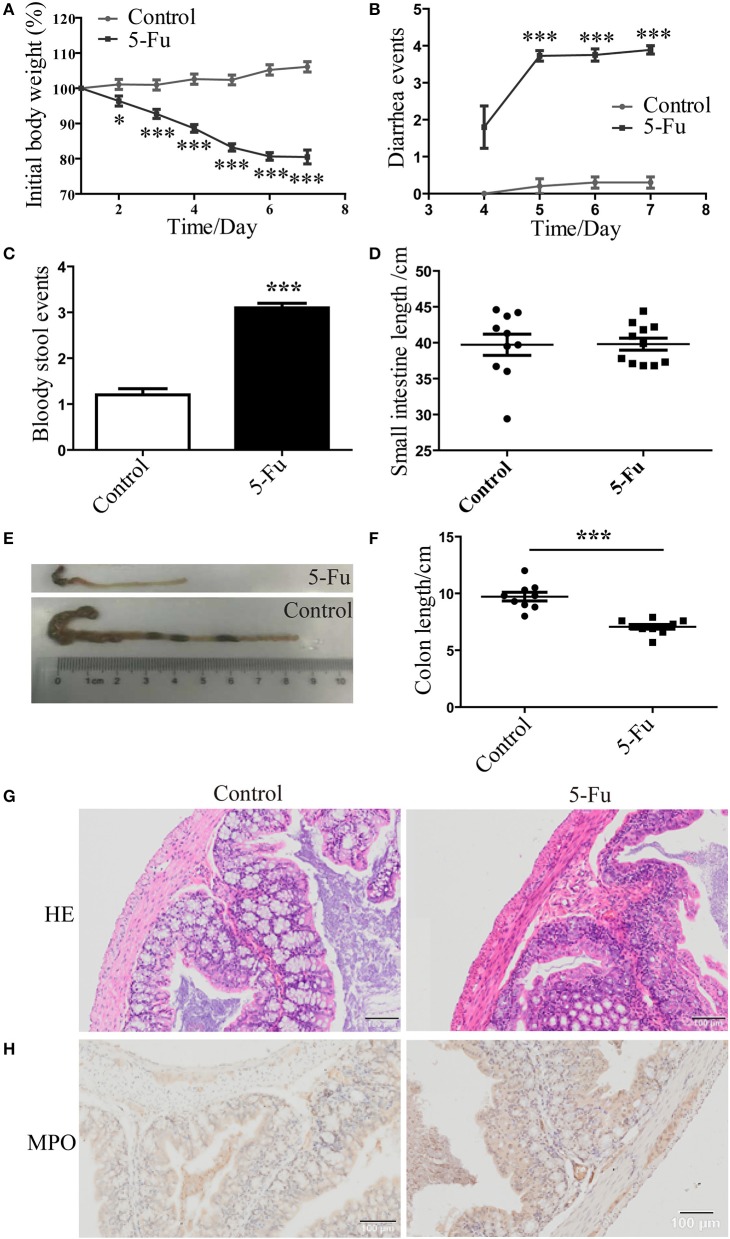
5-Fu induced mucositis and colon shortening in mice. **(A)** 5-Fu induced body weight changes. Data were plotted as percentage of initial body weight. **(B)** The occurrence of diarrhea. Data represented the evaluation scores of diarrhea. **(C)** The bloody stool events measured by BASO testing paper. **(D)** The small intestine length. **(E,F)** The colon length. **(G)** HE staining of colonic sections. **(H)** MPO staining of colonic sections. Values were expressed as mean ± S.E.M (*n* = 10/group). Data were analyzed by *t*-test. ^*^P < 0.05, ^***^P < 0.001 vs. control group.

Consistent with previous studies (Pereira et al., 2016), body weight of 5-Fu-treated mice was dramatically decreased from day 2 to day 7 after 5-Fu treatment (Figure 1A, *P* < 0.05 or *P* < 0.001), compared with the control mice. Meanwhile, severe diarrhea was found in 5-Fu group mice from day 5 to day 7 (Figure 1B, *P* < 0.001). At day 7, severe bloody stool was found in 5-Fu treated mice (Figure 1C). Shortened intestine indicates the increased contraction ability (Dou et al., 2013), while the shortened colon is closely associated with severe diarrhea. In our experiments, the small intestine length of 5-Fu treated mice was not changed compared to that of the control (Figure 1D). By contrast, the colon length of 5-Fu treated mice was significantly shortened (Figures 1E,F, *P* < 0.001) and the cecum of 5-Fu treated mice seemed to be smaller (Figure 1E). Moreover, 5-Fu treatment injured mucosal epithelium and disrupted crypt-villus structures, which was accompanied with enhance cellular infiltration (HE staining) and neutrophil (MPO staining) infiltration (Figures 1G,H).

### 5-Fu altered inflammatory cytokine and chemokine profiles

**Figure 2:**
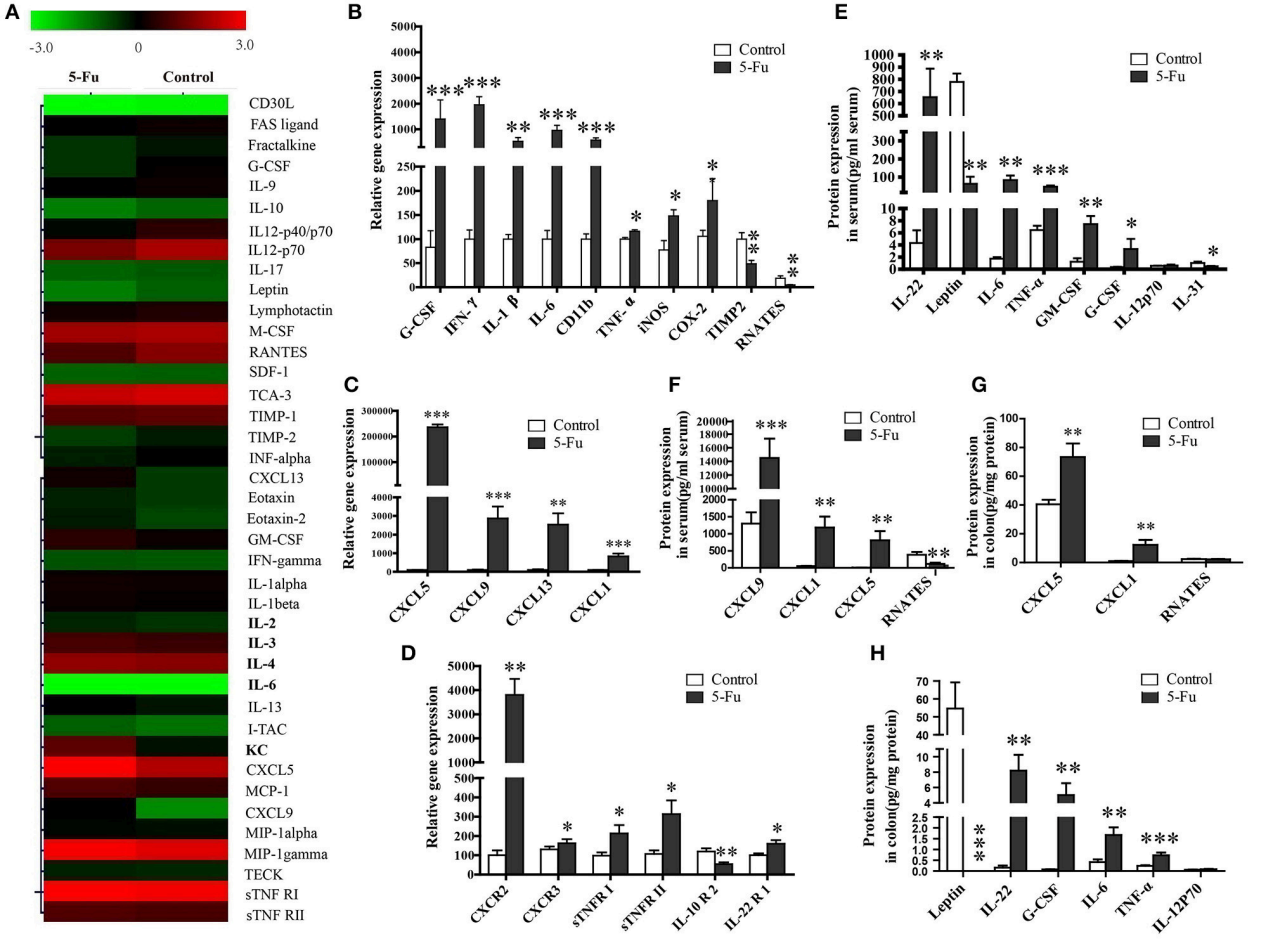
5-Fu induced the alteration of inflammatory chemokines/cytokines in colonic tissues or serum at protein and gene expression levels. **(A)** The alteration of inflammatory chemokines/cytokines in colonic tissure measured by protein chip analysis. **(B–D)** The inflammatory chemokines/cytokines gene expression measured by qPCR assay. **(E,F)** 5-Fu induced the alteration of inflammatory chemokines/cytokines in serum. **(G,H)** 5-Fu induced the alteration of inflammatory chemokines/cytokines in colonic tissues. Values were expressed as mean ± S.E.M (*n* = 10/group). Data were analyzed by *t*-test. ^*^P < 0.05, ^**^P < 0.01, ^***^P < 0.001 vs. control group.

Although previous studies exposed the alteration of several inflammatory factors in 5-Fu induced intestinal mucositis (Justino et al., 2014; Lu et al., 2015), the changed profile of the other inflammatory factors involved in the process has not been explored. In present study, a mouse inflammation antibody array (40 inflammatory factors) was employed to preliminarily examine the alteration of inflammatory factor profile. As shown in Figure 2A, compared to the control, 5-Fu seemed to elevate the protein levels of KC (CXCL1), LIX (CXCL5), MIG (CXCL9), B-lymphocyte chemoattractant (BLC), IL-6 and sTNFR I (>1.5-fold) but decrease that of G-CSF, IL-12p40/p70, RANETS, CD30L, Fractalkine, IL-10, IL12p70, Leptin, and TIMP-2 (>1.3-fold) in colonic tissues. In terms of mRNA expression of the cytokines/chemokines, 5-Fu treatment induced the mRNA expression of G-CSF, CD11b, iNOS, COX-2, interferon-γ (IFN-γ), IL-1β, IL-6, TNF-α, CXCL5, CXCL9, CXCL13, and CXCL1 (Figures 2B,C, *P* < 0.05, *P* < 0.01 or *P* < 0.001), but decreased that of TIMP2 and RNATES in colonic tissues. Moreover, as shown in Figure 2D, 5-Fu treatment modulated the mRNA expression of cytokine/chemokine receptors, as it up-regulated the mRNA expression of chemokine (C-X-C motif) receptor 2, 3 (CXCR2, CXCR3), sTNFR I, sTNFR II and interleukin-22 receptor 1 (IL-22R1), however, down-regulated that of interleukin-10 receptor 2 (IL-10R2). In order to further confirm the changes of inflammatory factors, the multiplex immunoassays and ELISA assay were performed, respectively. As illustrated in Figures 2E,F, in serum of 5-Fu-induced mice, the protein levels of CXCL9, CXCL1 (KC), CXCL5, IL-22, IL-6, TNF-α, GM-CSF, and G-CSF were significantly increased (*P* < 0.05, *P* < 0.01, or *P* < 0.001), but that of RNATES, Leptin, and IL-31 were significantly decreased (*P* < 0.05, *P* < 0.01, or *P* < 0.001). Similarly, in colonic tissues of 5-Fu-induced mice, the protein levels of IL-22, G-CSF, IL-6, TNF-α, CXCL1 (KC), and CXCL5 were significantly elevated (Figures 2G,H, *P* < 0.01 or *P* < 0.001), while that of Leptin was significantly reduced (*p* < 0.001). By contrast, protein level of IL-12p70 did not change in both serum and colonic tissues.

### 5-Fu modulated the expression of tight junctions (TJ) and adhesion proteins

**Figure 3:**
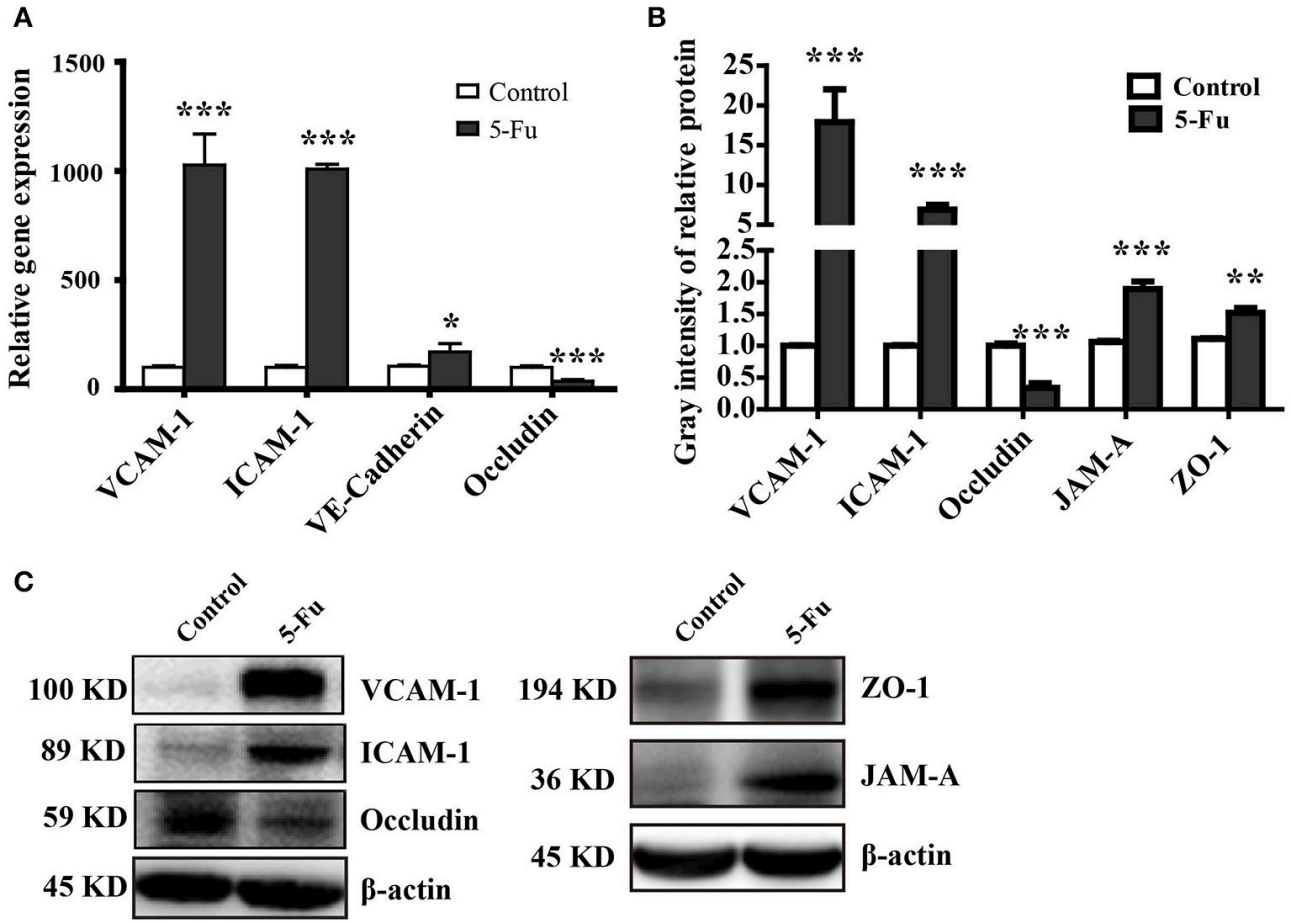
5-Fu regulated the expression of tight junction and adhesion molecules in colonic tissues. **(A)** 5-Fu regulated gene expression of tight junction and adhesion molecule measured by qPCR analysis (*n* = 6/group). **(B,C)** 5-Fu regulated protein expression of occludin, VCAM-1, ICAM-1, JAM-A, and ZO-1(*n* = 3-4/group). β-actin was used as the endogenous reference. Values were expressed as mean ± S.E.M. Data were analyzed by *t*-test. ^*^P < 0.05, ^**^P < 0.01, ^***^P < 0.001 vs. control group.

Tight junction supports the integral intestinal epithelial barrier structure and barrier function, which is disrupted under inflammation (Capaldo et al., 2017; Chang et al., 2017). Adhesion molecules mediate the attachment of lymphocytes, neutrophils and inflammatory cells to the endothelial cells under inflammatory condition (Erbeldinger et al., 2017; Kim et al., 2017). As shown in Figure 3, 5-Fu treatment induced significant mRNA expression of adhesion molecules, VCAM-1, ICAM-1, and VE-Cadherin (*P* < 0.001, *P* < 0.001, and *P* < 0.05) as well as the protein expression of VCAM-1 and ICAM-1 (*P* < 0.001) in colon. However, in terms of tight junction proteins, 5-Fu decreased mRNA and protein expression of occludin (*P* < 0.001, *P* < 0.001). But 5-Fu increased the protein level of JAM-A and ZO-1 (*P* < 0.001 and *P* < 0.01).

### 5-Fu activated MAPK and NF-κB pathway signaling

**Figure 4:**
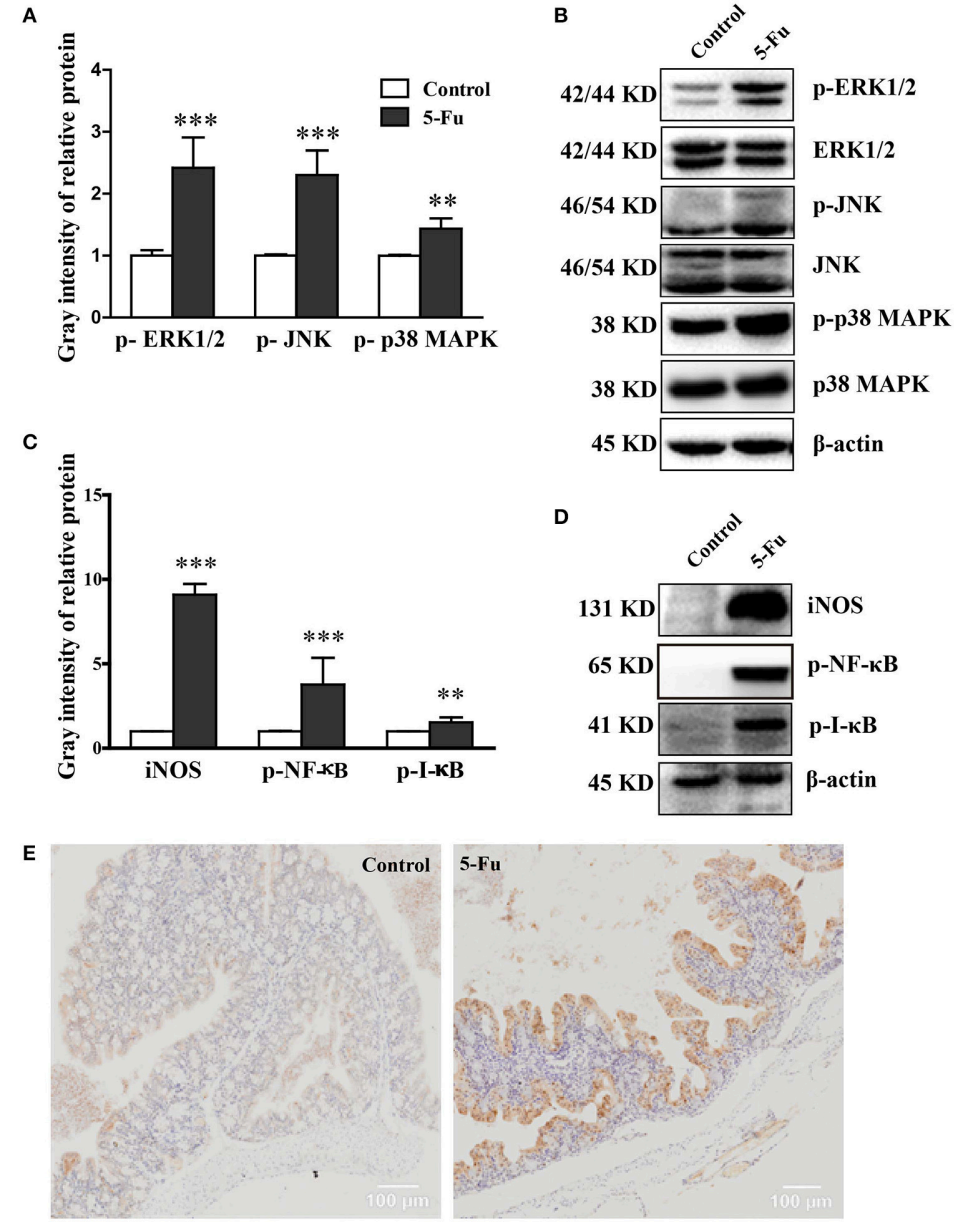
5-Fu activated NF-κB and MAPK signaling pathways in colonic tissues. **(A,B)** 5-Fu treatment enhanced the protein expression of p-ERK1/2, p-JNK, and p-p38 MAPK. **(C,D)** 5-Fu treatment elevated the protein expression of iNOS, p-NF-κB, and p-I-κB. **(E)** 5-Fu treatment increased the expression of activated NF-κB in the colonic epithelial cells. Values were expressed as mean ± S.E.M (*n* = 3/group). Data were analyzed by *t*-test. ^**^P < 0.01, ^***^P < 0.001 vs. control group.

MAPK and NF-κB pathways are closely associated with inflammation (Park et al., 2013). To determine whether MAPK and NF-κB pathways were involved in 5-Fu-induced colonic mucositis, we further assessed the effect of 5-Fu treatment on the activation of signaling molecules, including ERK1/2, JNK, p38 MAPK, IκB and NF-κB. As shown in Figure 4, 5-Fu enhanced the phosphorylation of ERK1/2, JNK, p38 MAPK, IκB and NF-κB as well as the protein expression of iNOS in the colon (*P* < 0.001, or *P* < 0.01). Moreover, 5-Fu treatment increased the expression of activated NF-κB in the intestinal epithelial cells (Figure 4E). All of these results indicated that 5-Fu treatment resulted in the activation of MAPK and NF-κB signaling pathways.

### 5-Fu altered bacterial diversity and community composition

**Figure 5:**
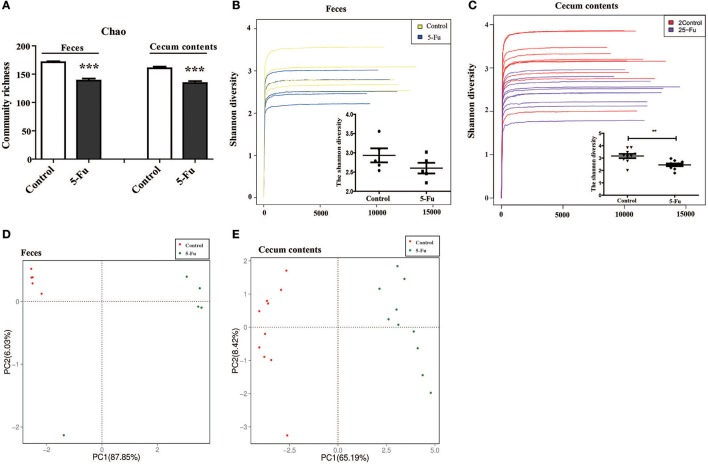
5-Fu decreased the richness and diversity of gut microbiota in feces and cecum contents of mice. **(A)** 5-Fu decreased the richness of gut microbiota in feces and cecum contents. Chao is an estimator of the community richness. **(B,C)** 5-Fu decreased the diversity of gut microbiota in feces (**B**, Shannon index curves and Shannon diversity histogram) and cecum contents (**C**, Shannon index curves and Shannon diversity histogram). **(D,E)** The unweighted UniFrac PCoA results of feces **(D)** and cecum contents **(E)** for beta-diversity at OTUs level. Values were expressed as mean ± S.E.M (*n* = 5/group, feces; *n* = 10/group, cecum contents). Data were analyzed by *t*-test. ^**^P < 0.01, ^***^P < 0.001 vs. control group.

Gut microbiota has been indicated in inflammatory bowel disease (Terán-Ventura et al., 2014; Patel et al., 2016). Alteration of gut microbiota composition may affect the function of mucosal immune system, resulting in the intestinal inflammation (Autenrieth and Baumgart, 2017; Etienne-Mesmin et al., 2017; Holleran et al., 2017). Therefore, to clarify the change of gut microbiota of 5-Fu treated mice, the diversity and composition of gut microbiota in cecum contents and feces were analyzed by Miseq sequencing. The Chao community richness and Shannon diversity were used to estimate within-community diversity (α-diversity). Sequencing of 16S rRNA gene V3-V4 region of gut microbiota showed that 5-Fu greatly decreased the community richness of microbiota in both feces and cecum contents, compared with the controls (Figure 5A, *P* < 0.001). It significantly decreased the Shannon diversity in cecum contents but not that in feces of mice (Figures 5B,C, *P* < 0.01). Unweighted UniFrac PCoA analysis demonstrated that there was a significant difference between control and 5-Fu treated mice regarding beta-diversity at OTUs level (Figures 5D,E). These results indicated that 5-Fu treatment led to the richness and diversity loss in the bacterial community, especially in cecum contents.

**Figure 6:**
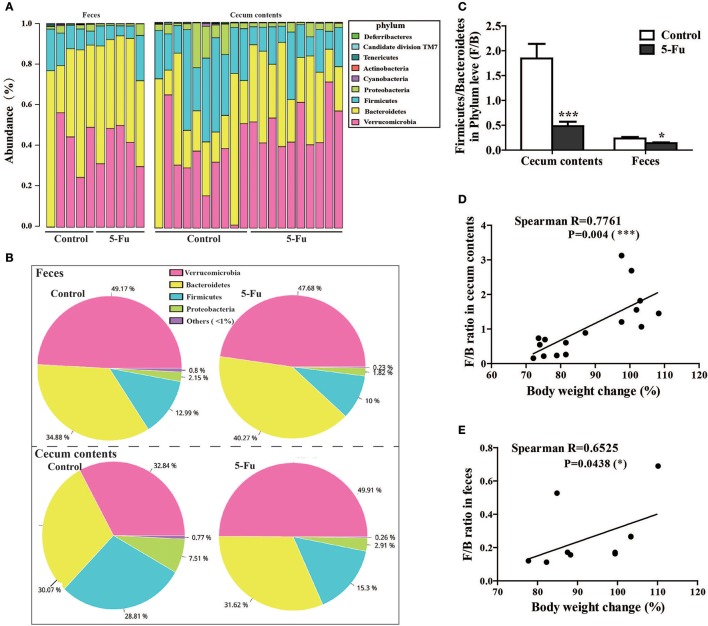
5-Fu treatment changed the gut microflora community composition at phylum level in feces and cecum contents of mice. **(A)** The bar diagram of bacterial community distribution in cecum contents (*n* = 10/group) and feces (*n* = 5/group) at phylum level, respectively. **(B)** The pie charts depicting the mean relative abundance for feces and cecum contents at phylum level, respectively. **(C)** 5-Fu treatment down-regulated the ratio of F/B in cecum contents and feces. **(D)** The correlation between body weight changes and F/B ratio in cecum contents. **(E)** The correlation between body weight changes and F/B ratio in feces. Values were expressed as mean ± S.E.M (*n* = 5/group, feces; *n* = 10/group, cecum contents). Data were analyzed by *t*-test. ^*^P < 0.05, ^***^P < 0.001 vs. control group.

The four major phyla in the feces and cecum contents were Bacteroidetes, Verrucomicrobia, Firmicutes, and Proteobacteria (Figures 6A,B, Table S1), among which Bacteroidetes and Verrucomicrobia were the relatively abundant ones. 5-Fu treatment remarkably decreased the relative abundance of Firmicutes, Proteobacteria, and Cyanobacteria at phyla level in feces (*P* < 0.05 or *P* < 0.01). However, 5-Fu increased the abundance of Verrucomicrobia (*P* < 0.05), although it also reduced that of Firmicutes and Cyanobacteria (*P* < 0.01) in cecum contents. In addition, 5-Fu significantly decreased the ratio of Firmicutes/Bacteroidetes (F/B) in cecum contents and feces (Figure 6C, *p* < 0.001, *p* < 0.05). Further correlation analysis (Figures 6D,E) showed that F/B ratio positively correlated with body weight change (Spearman's *R* = 0.7761, *P* < 0.001 in cecum; Spearman's *R* = 0.6525, *P* < 0.05 in feces). More information about gut microbiota in cecum contents and feces could be found in Supplementary Data (Tables S1–12).

### Disturbed gut microbiota was involved in body weight loss and colon shortening in 5-Fu induced colonic mucositis

**Figure 7:**
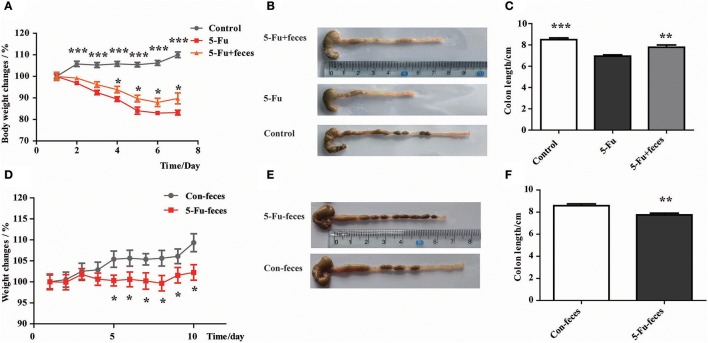
Disturbed gut microbiota resulted in body weight loss and colon shortening in 5-Fu treated mice. **(A)** Normal fecal microbiota transplantation inhibited 5-Fu induced body weight loss. Data plotted as percentage of initial body weight. **(B,C)** The effect of normal fecal microbiota transplantation on colon length. Values were expressed as mean ± S.E.M (*n* = 7/group). Data were analyzed by *t*-test. ^*^P < 0.05, ^**^P < 0.01, ^***^P < 0.001 vs. 5-Fu group. **(D)** Fecal microbiota transplantation from 5-Fu treated mice induced body weight loss in mice pretreated with vancomycin. Data were plotted as percentage of initial body weight. **(E,F)** The effect of fecal microbiota transplantation from 5-Fu treated mice on colon length in vancomycin pretreated mice. Values were expressed as mean ± S.E.M (*n* = 10/group). Data were analyzed by *t*-test. ^*^P < 0.05, ^**^P < 0.01 vs. control group.

As shown in Figure 7A, from day 4, fecal transplantation significantly rescued the body weight loss of mice induced by 5-Fu treatment (*P* < 0.05). Furthermore, at day 7, fecal microbiota transplantation prevented the shortening of colon induced by 5-Fu treatment (Figures 7B,C) (*P* < 0.01). In another experiment, to assess the effect of fecal microbiota on 5-Fu induced colonic mucositis, the vancomycin-pretreated mice were transplanted with feces from Control and 5-Fu group mice, respectively. As shown in Figures 7D–F, compared to mice transplanted with normal feces, mice transplanted with feces from 5-Fu treated mice showed significant body weight loss and shortened colon. These results implicated that disturbed gut microbiota contributed to the induction of intestinal mucositis in 5-Fu treated mice.

## Discussion

Although previous studies have indicated that gut microbiota plays an important role in 5-Fu induced gastrointestinal mucositis (Stringer et al., 2009b; Chang et al., 2012; Gao et al., 2014), however, none of them described the causal relationship in a systemic way. In present study, we analyzed the alteration of gut microbiota and inflammatory cytokine/chemokine profiles with relatively systemic methods. Our findings showed that, besides small intestine mucositis, 5-Fu also induced colonic mucositis. Both gut microbiota and inflammatory cytokine/chemokine profiles were altered significantly, which was accompanied with mucosal barrier disruption and inflammatory signaling pathway activation. Further studies revealed that fecal transplant from healthy mice alleviated the severity of colonic mucositis, while that from 5-Fu treated mice seemed to induce significant symptoms of colonic mucositis. Our results indicated that the recovery of homeostasis of gut microbiota by fecal transplantation might facilitate the relief of gastrointestinal mucositis induced by 5-Fu.

Pro-inflammatory cytokines and anti-inflammatory factors play a critical role in inflammatory bowel diseases (Stringer et al., 2009b). The expression levels of IL-6, TNF-α, IL-1β, IFN-γ, CXCL1 were shown to increase in small intestine of 5-Fu-induced intestinal mucositis mice (Soares et al., 2011, 2013; Chang et al., 2012; Yasuda et al., 2013; Yeung et al., 2015). In present study, IL-6, TNF-α, IL-1β were remarkably increased in serum and/or colon tissue at both mRNA and protein levels in 5-Fu induced colonic mucositis mice. Meanwhile, 5-Fu elevated the levels of IFN-γ, G-CSF, GM-CSF, and CD11b, while decreased that of RNATES and IL-31. Interestingly, leptin, a hormone produced and secreted by adipose tissue, muscle and stomach, was also detected in colonic tissue. And, 5-Fu could significantly decrease leptin both in serum and colonic tissues. Leptin treatment has been shown to promote intestinal recovery and enhance enterocyte turnover in a rat model of methotrexate-induced mucositis (Sukhotnik et al., 2009). The decreased leptin in serum and colon might partly reflect the excerbation of colonic mucositis induced by 5-Fu. CXCL9 treatment has been disclosed to attenuate 5-Fu induced mucositis (Han et al., 2011), however, it exacerbates 5-Fu induced acute intestinal damage (Lu et al., 2015). Therefore, further investigation is needed to clarify the effect of CXCL9 on 5-Fu induced mucositis. So far the role of CXCL5 in 5-Fu induced mucositis has not been elucidated yet. CXCL13 mediates T cell recruitment and participates in the regulation of inflammatory response (Hui et al., 2015). IL-22 produced by T cells and NK cells participates in tumorigenesis and tumor progression, and mediates chemoresistance (Wu et al., 2014), which is enhanced in colon of 5-Fu induced mice (Sakai et al., 2013). In present study, 5-Fu treatment significantly modulated the levels of CXCL5, CXCL9, CXCL13, and IL-22 in serum and/or colonic tissues. Moreover, 5-Fu increased gene expression of CXCR2 (receptor of CXCL1, CXCL5), CXCR3 (receptor of CXCL9), sTNFRI and sTNFRII (receptors of TNF-α), and IL-22R1 (receptor of IL-22), while reduced that of IL-10R2 (receptor of IL-10) in colonic tissue. All of these results implicated that 5-Fu induced colonic mucositis along with significant inflammatory responses.

TJs maintain the intestinal mucosal barrier (Yang et al., 2015). Reduction of TJs expression always indicates the increased intestinal epithelial permeability (Park et al., 2015; Yang et al., 2015). Chemotherapeutic drug could increase intestinal epithelial barrier permeability via reducing protein expression of TJs (Beutheu Youmba et al., 2012). Inflammatory infiltration is a characteristic of mucositis, which is triggered by the increased adhesion molecules in intestinal endothelia that attract the circulating inflammatory cells including neutrophils, T lymphocyte cells, B lymphocyte cells to gather in the inflammatory sites (Erbeldinger et al., 2017; Kim et al., 2017). The inflammatory cells further accelerate the modification of tight junction, thereby increase intestinal permeability leading to the disruption of mucosal barrier (Leocádio et al., 2015). The elevated pro-inflammatory cytokines induced by 5-Fu have been shown to account for the loss of tight junction proteins of small intestine, such as occludin and claudin-1, and result in diarrhea (Patel et al., 2016). In colonic tissues, our results demonstrated that 5-Fu treatment disrupted tight junction as the expression of occludin was down-regulated at both mRNA and protein levels. Surprisingly, ZO-1 protein was upregulated by 5-Fu in our study. It is well-known that ZO-1 is a cytoplasmic scaffolding protein, which is breakdown or redistributed under TNF-α-induced inflammatory condition (Chen et al., 2017; Watari et al., 2017). However it did not show significant change in mucosa of 5-Fu-induced small intestine (Song et al., 2013) or irinotecan-induced gut toxicity (Wardill et al., 2014), suggesting that its specific role on mucosal barrier under these inflammatory conditions. We don't know whether there is a compensatory mechanism in ZO-1, because of the decreased expression of occludin in 5-Fu induced colonic mucositis. Meanwhile, adhesion proteins such as ICAM-1, VCAM-1, JAM-A, and VE-Cadherin were increased by 5-Fu. These results indicated that 5-Fu treatment might increase the colonic epithelial barrier permeability through decreasing TJ proteins and up-regulating adhesion proteins to recruit inflammatory cells to colonic epithelium, and then enhance the translocation of gut microbiota in the mucosa to promote the inflammation.

NF-κB and MAPK pathways can be activated by many inflammatory chemokines/cytokines, which may result in a pro-inflammatory chemokines/cytokines positive feedback (Zimmerman et al., 2008; Tung et al., 2011; Chang et al., 2012; Jiang et al., 2012; Song et al., 2013; Candela et al., 2014; Dou et al., 2014; Yeung et al., 2015). And their activation in the small intestine has been shown to be involved in 5-Fu induced mucositis (Liu et al., 2013). Also, methotrexate (MTX) treatment increased intestinal permeability partially related to the decreased TJs protein expression through MAPK and NF-κB pathways (Beutheu Youmba et al., 2012). In agreement with the report, in our study, enhanced phosphorylation of NF-κB and MAPK pathway molecules were also found in colonic tissue of 5-Fu treated mice, indicating their active participation in regulating expression of tight junction proteins and colonic proinflammatory cytokines/chemokines in the pathogenesis of 5-Fu induced colonic mucositis.

Disturbed gut microbiota, in either diversity or abundance, has been found to play an important role in the pathological development of inflammatory bowel diseases (Juste et al., 2014; Rangel et al., 2016). At genus level, 5-Fu treatment has been shown to decrease *Clostridium, Lactobacillus, Streptococcus* and *Enterococcus* and increase *Escherichia* in rat jejunum or colon (Stringer et al., 2007, 2009b). Different from the findings in rat, in present study, 5-Fu significantly decreased *Odoribacter, Candidatus Saccharimonas* and *Marvinbryantia*, and increased *Helicobacter* and *Thalassospira* in mouse feces. Moreover, 5-Fu significantly changed the abundance of *Blautia, Alistipes, Coprococcus, Roseburia, Akkermansia, Bilophila, Candidatus Saccharimonas*, and *Mucispirillum* in mouse cecum contents. The difference between our findings and previous reports might reflect the variable microbiota profiles affected by multiple factors such as environment, diet, gender, age, and species. At phylum level, microbiota low in Firmicutes has been disclosed to enhance the intestinal sensitivity to inflammation (Natividad et al., 2015). Decreased abundance of Ruminococcaceae and Lachnospiraceae families belong to Firmicutes phylum is found to be associated with inflammatory states (Knip and Siljander, 2016). On the contrary, cocktail of Ruminococcaceae and Lachnospiraceae families can efficiently reverse experimental colitis induced by dextran sodium sulfate (DSS) (Natividad et al., 2015). The ratio of Firmicutes/Bacteroidetes seems to be important for the maintenance of physiological state as the relative abundance of Firmicutes/Bacteroidetes influences body weight of animals, especially in metabolic diseases (Turnbaugh et al., 2006; Kassinen et al., 2007; Remely et al., 2014). Proteobacteria has been shown to play a crucial and active role in overall gut metabolism and host response despite their low abundance (Pérez-Cobas et al., 2013). Cyanobacteria inhibits inflammation by production of anti-inflammatory pitinoic acids B and C (Montaser et al., 2013). On the contrary, Verrucomicrobia appears to contribute to inflammation as their abundance bloomed in mice treated with DSS (Nagalingam et al., 2011). In present study, 5-Fu reduced the richness and diversity of gut microbiota, the relative abundance of Lachnospiraceae and Ruminococcaceae families (Tables S4, S10) accompanied with a lower ratio of Firmicutes/Bacteroidetes. Moreover, 5-Fu lessened the relative abundance of Proteobacteria, Candidate division TM7 and Cyanobacteria while increased that of Verrucomicrobia and Actinobacteria. These results implicated the active involvement of the microbiota in 5-Fu induced colonic mucositis. We also found that there was significant positive correlation between body weight changes and F/B ratio in feces and cecum contents. Our further results showed that fecal transplantation from normal mice could partly reverse the body weight loss and colon length decrease of 5-Fu treated mice. Moreover, feces from 5-Fu treated mice could also result in body weight loss and colon length decrease in normal mice pretreated with vancomycin. These results demonstrated that gut microbiota dysfunction at least partly accounted for the mucositis induced by 5-Fu. And the increased colonic epithelial barrier permeability induced by 5-Fu, would promote the translocation of gut bacteria in the intestinal mucosa, then to increase the inflammatory response (Escobedo et al., 2014; Mayer et al., 2015; Severance et al., 2016; Leung and Yimlamai, 2017).

In summary, our results indicated that 5-Fu induced mucositis might be partly mediated by the disturbance of gut microbiota. Since modulation gut microbiota by administration of probiotics or certain gut microbiota metabolite seemed to benefit 5-Fu-induced mucositis (Justino et al., 2014; González-Sarrías et al., 2015; An and Ha, 2016; Flórez et al., 2016), our findings provided potential novel therapeutic strategy for patients suffered from 5-Fu induced intestinal mucositis by manipulation of specific gut microbiota.

## Author contributions

HS and XWu designed all the experiments, analyzed data and wrote the paper, and the performances of HS and XWu were equal in this study. HL and LL carried out the main experiments, and the performances of HL and LL were equal in this study; XWang, LQ, PW, SQ, and HWu performed parts of experiments. FH, and BZ provided valuable suggestions for this study and helped to draft the manuscript. All authors read and approved the final manuscript.

### Conflict of interest statement

The authors declare that the research was conducted in the absence of any commercial or financial relationships that could be construed as a potential conflict of interest.

## Abbreviations

5-Fu: 5-fluorouracil
MPO: myeloperoxidase
IFN-γ: interferon-γ
IL-1/6: interleukin-1/6
TNF-α: tumor necrosis factor-α
CXCL1/5/9/13: chemokine (C-X-C motif) ligand 1/5/9/13
IL-22 R1: interleukin-22 receptor 1
IL-12 R2: interleukin-12 receptor 2
VCAM-1: vascular cell adhesion molecule-1
ICAM-1: intercellular adhesion molecule-1
G-CSF: granulocyte colony stimulating factor
GM-CSF: granulocyte-macrophage colony stimulating factor
BSA: bovine serum albumin
MAPK: mitogen activated protein kinase
ERK: extracellular signal-regulated kinase
SAPK/JNK: stress activated protein kinase/jun N-terminal kinase
p-ERK: phosphorylated extracellular signal-regulated kinase
p-JNK: phosphorylated jun N-terminal kinase
iNOS: inducible NO synthase
ZO-1: zonula occludens-1
JAM-A: junctional adhesion molecule-A.
