# Supplementary material for: Alteration of Gut Microbiota and Inflammatory Cytokine/Chemokine Profiles in 5-Fluorouracil Induced Intestinal Mucositis
Source: Front Cell Infect Microbiol. 2017 Oct 26;7:455. doi: 10.3389/fcimb.2017.00455 (PMC5662589; doi:10.3389/fcimb.2017.00455)
Supplement: Supplementary file 1 [file Presentation1.pdf]

Alteration of gut microbiota and inflammatory cytokine/chemokine profiles in 5-fluorouracil induced intestinal mucositis

Hong-Li Li#, Lan Lu#, Xiao-Shuang Wang, Li-Yue Qin, Ping Wang, Shui-Ping Qiu, Hui Wu, Fei Huang, Bei-Bei Zhang, Hai-Lian Shi*, Xiao-Jun Wu*

Shanghai Key Laboratory of Compound Chinese Medicines, The Ministry of Education (MOE) Key Laboratory for Standardization of Chinese Medicines, Institute of Chinese Materia Medica, Shanghai University of Traditional Chinese Medicine, Shanghai, 201203, China

* Corresponding author at: Institute of Chinese Materia Medica, Shanghai University of Traditional Chinese Medicine, 1200 Cailun Road, Shanghai, 201203, China. Tel.: +86 21 51322577; Fax: +86 21 51322578; E-mail address: shihailian2003@163.com (HL Shi); xiaojunwu320@126.com (XJ Wu).

#These authors contributed equally to this work.

Running title: Gut microbiota and 5-Fu induced intestinal mucositis

Table S1 The composition of the flora community at the level of phylum in feces (T-test)

Composition	Control	5-Fu	P value
Verrucomicrobia	0.4356±0.06799	0.4027±0.04211	0.6799
Bacteroidetes	04231±0.08080	0.4768±0.02934	0.5153
Firmicutes	0.1452±0.02328	0.06948±0.01062	0.0253 *
Proteobacteria	0.02551±0.005710	0.008886±0.0003568	0.0272 *
Cyanobacteria	0.002693±0.0007608	0.0001695±0.0001695	0.0084 **
Actinobacteria	0.0002417±0.00009627	0.0008819±0.00008486	0.0016 **
Candidate division TM7	0.003212±0.0006694	0.0006372±0.0002150	0.0049 **
Deferribacteres	0.00009818±0.00007302	0.0002962±0.00007995	0.1172
Tenericutes	0.00238574		

Table S2 The composition of the flora community at the level of class in feces (T-test)

Composition	Control	5-Fu	P value
Verrucomicrobiae	0.4356±0.06799	0.4027±0.04211	0.6799
Bacteroidia	0.4917±0.09291	0.4768±0.02934	0.8818
Clostridia	0.1229±0.02367	0.09086±0.03353	0.4573
Eosilonproteobacteia	0.001462±0.0003436	0.004515±0.001169	0.0367 *
Grammaproteobacteria	0.006617±0.001381	0.003769±0.001427	0.2018
Alphaproteobacteria	0.01415±0.005350	0.0007946±0.0007084	0.0264 *
Deltaproteobacteria	0.003296±0.0005752	0.002056±0.0005849	0.1789
Erysipelotrichia	0.005560±0.002444	0.007786±0.002730	0.5603
Bacilli	0.001445±0.0005651	0.0004300±0.0002130	0.1722
Deferribacteres	0.0005289±0.0004344	0.0006517±0.0003609	0.8333
Unclassified	0.004468±0.001312	0.001754±0.0001729	0.0861
Nollocutes	0.00192405		
Actinobacteri		0.00020656	

Table S3 The composition of the flora community at the level of order in feces (T-test)

Composition	Control	5-Fu	P value
Verrucomicrobiales	0.3488±0.1015	0.4027±0.04211	0.6373
Bacteroidales	0.4917±0.09291	0.4768±0.02934	0.8818
Clostridiales	0.1229±0.02367	0.09086±0.03353	0.4573
Campylobacterales	0.001462±0.0003436	0.004515±0.001169	0.0367 *
Enterobacteriales	0.004595±0.001510	0.002709±0.001111	0.3439
Rhodospirillales	0.01134±0.005009	0.0007946±0.0007084	0.0706
Desulfovibrionales	0.003296±0.0005752	0.002056±0.0005849	0.1789
Erysipelotrichales	0.005560±0.002444	0.007786±0.002730	0.5603
Lactobacillales	0.001445±0.0005651	0.001322±0.0009072	0.9113
Deferribacterales	0.001541±0.0005151	0.0004616±0.0002204	0.0.0700
Unclassified	0.006959±0.002331	0.001013±0.0001832	0.0346 *
Coriobacterales	0.0005489±0.0003161	0.0006753±0.0001439	0.7253
Pseudomonadales	0.0007853±0.0003274	0.001060±0.0003241	0.5669

Table S4 The composition of the flora community at the level of family in feces (T-test)

Composition	Control	5-Fu	P value
Verrucomicrobiaceae	0.3934±0.07540	0.4287±0.04275	0.6807
Provotellaceae	0.1128±0.05667	0.1216±0.03597	0.8990
Unclassifed	0.3037±0.06738	0.2634±0.02136	0.5841
Lachnospiraceae	0.07114±0.01841	0.01895±0.004308	0.0435 *
Helicobacteraceae	0.001462±0.0003436	0.004515±0.001169	0.0367 *
Ruminococcaceae	0.04628±0.01201	0.04308±0.01064	0.8467
Bacteroidaceae	0.01186±0.003274	0.05655±0.01451	0.0170 *
Rikenellaceae	0.04755±0.01876	0.02050±0.002595	0.1911
Enterobacteriaceae	0.005657±0.001386	0.002709±0.001111	0.1363
Porphyromonadaceae	0.02587±0.008819	0.01738±0.003607	0.3988
Rhodospirillaceae	0.01415±0.005350	0.0007946±0.0007084	0.0264 *
Peptococcaceae	0.001619±0.0004530	0.0008538±0.00005141	0.1316
Desulfovibrionaceae	0.003296±0.0005752	0.009170±0.007128	0.4352
Erysipelotrichaceae	0.005560±0.002444	0.007786±0.002730	0.5603
Lactobacillaceae	0.001445±0.0005651	0.001322±0.0009072	0.9113
Coriobacteriaceae	0.0005489±0.0003161	0.0006753±0.0001439	0.7253
Defluviitaleaceae	0.0007847±0.0005116	0.00009102±0.00003922	0.2134
Deferribacteraceae	0.0005289±0.0004344	0.0006517±0.0003609	0.8333
Moraxellaceae	0.0007853±0.0003274	0.001060±0.0003241	0.5669
Propionibacteriaceae		0.00020656	

Table S5 The composition of the flora community at the level of genus in feces (T-test)

Composition	Control	5-Fu	P value
Akkermansia	0.3488±0.1015	0.4027±0.04211	0.6373
Alloprevotella	0.0957±0.04885	0.1190±0.03555	0.7196
Unclassifed	0.4189±0.08401	0.3219±0.03140	0.3112
Blautia	0.04596±0.01108	0.02530±0.01775	0.3524
Helicobacter	0.001462±0.0003436	0.004515±0.001169	0.0367 *
Anaerotruncus	0.002597±0.0007430	0.003980±0.001194	0.3542
Bacteroides	0.01186±0.003274	0.05655±0.01451	0.0170 *
Odoribacter	0.01156±0.003767	0.0002374±0.002374	0.0171 *
Parabacteroides	0.01432±0.005142	0.01714±0.003700	0.6674
Ruminococcus	0.0008885±0.0002775	0.006665±0.002870	0.0801
Alistipes	0.01134±0.005009	0.0007946±0.0007084	0.0706
Desulfovibrio	0.01631±0.004094	0.01467±0.001775	0.7231
Oscillibacter	0.002673±0.0004783	0.008237±0.006620	0.4262
Rikenella	0.002713±0.0009053	0.002134±0.0005740	0.6035
Bilophila	0.003474±0.0009356	0.005814±0.001140	0.1513
Coprococcus	0.0006230±0.0001093	0.0009331±0.0005116	0.5697
Roseburia	0.001666±0.0006232	0.002461±0.002059	0.7213
Lactobacillus	0.0004067±0.0001320	0.001268±0.0007312	0.2330
Enterorhabdus	0.001445±0.0005651	0.001322±0.0009072	0.9113
Candidatus Saccharimonas	0.0005489±0.0003161	0.0006753±0.0001439	0.7253
Candidatus Arthromitus	0.002690±0.0007351	0.0006372±0.0002150	0.0279 *
Marvinbryantia	0.0009203±0.0002350	0.0001595±0.00009371	0.0169 *
Mucispirillum	0.0001703±0.00006582	0.001123±0.0004301	0.0600
Turicibacter	0.0005289±0.0004344	0.0006517±0.0003609	0.8333
Acinetobacter	0.0008098±0.0003323	0.00007907±0.00003480	0.0602
Propionnibacterium	0.0007853±0.0003274	0.001060±0.0003241	0.5669
Thalassospira		0.00020656	

Table S6 The composition of the flora community at the level of species in feces (T-test)

Composition	Control	5-Fu	P value
Unclassified	0.7238±0.06837	0.6403±0.02355	0.2815
Uncultured bacyeroidales bacterium	0.2062±0.04252	0.2685±0.02144	0.2272
Helicobacter ganmani	0.001051±0.0003576	0.002833±0.0007646	0.0678
Anaerotruncus sp. G3	0.002067±0.0006848	0.003487±0.0009402	0.2568
Gut metagenome	0.0307932		
Bacteroides vulgatus(ATCC 8428)	0.0002376±0.00005330	0.03796±0.01395	0.0269 *
Eubacterium coprostanoligenes	0.00515534		
Firmicutes bacterium ASF500	0.003883±0.001304	0.002433±0.0005846	0.3402
Parabacteroides goldsteinii	0.0003321±0.0001730	0.009337±0.002903	0.0147 *
Clostridium papyrosolvens	0.01048±0.009080	0.002972±0.001697	0.4397
Mouse gut metagenome	0.0003874±0.0001732	0.001335±0.0007863	0.2730
Bacteroides acidifaciens	0.004226±0.0006173	0.01081±0.002800	0.0508
Lachnospiraceae bacterium 610	0.0000327765		
Uncultured rikenellaceae bacterium	0.0005278±0.0001933	0.005373±0.001754	0.0252 *
Bacteroids uniformis	0.00005299±0.00003684	0.005662±0.002817	0.0816
Desulfovibrio sp. ABHU1SB	0.0005155±0.0004584	0.0004687±0.0002772	0.9326
Unidentified rumen bacterium RFN71	0.001409±0.0004383	0.002340±0.0009056	0.3817
Clostridium sp. ABHU1SB	0.00007113±0.00005451	0.0003307±0.0001612	0.1656
Bacteroides bacterium ph8	0.0002058±0.00008006	0.0003478±0.0001581	0.4462
Lachnospiraceae bacterium 10-1	0.0006687±0.0004080	0.0001456±0.0001066	0.2500
Dorea sp. 5-2	0.0005632±0.0001401	0.0006022±0.0001472	0.8524
Clostridium sp. Culture-41	0.0009228±0.0002589	0.0001941±0.0001198	0.0339 *
Clostridiales bacterium CIEAF 020	0.00274196		
Clostridiales bacterium CIEAF 012	0.0001703±0.00006582	0.001123±0.0004301	0.0600
Unclutured clostridiales bacterium		0.000437517	
Unclutured rumen bacterium	0.001691±0.0009883	0.0007946±0.0007084	0.4819
Bacteroides stercoris(ATCC 43183)	0.0001507±0.00001942	0.0002309±0.0001230	0.5376
Clostridium sp. Culture-27	0.000163524		
Clostridium leptum	0.0002351±0.00004341	0.0007264±0.0003916	0.2477
Lactobacillus murinus	0.001206±0.0005052	0.0009966±0.0008175	0.8327
Clostridium sp. ATCC 29733		0.000525516	

Table S7 The composition of the flora community at the level of phylum in cecum contents (T-test)

Composition	Control	5-Fu	P value
Verrucomicrobia	0.3007±0.06402	0.4991±0.03395	0.0135 *
Bacteroidetes	0.3284±0.07683	0.3162±0.03796	0.8877
Firmicutes	0.2881±0.03875	0.1530±0.02468	0.0088 **
Proteobacteria	0.05923±0.01809	0.02911±0.005661	0.1149
Cyanobacteria	0.001784±0.0004976	0.00005597±0.00003290	0.0019 **
Actinobacteria	0.0002655±0.00005982	0.001087±0.0003288	0.0243 *
Candidate division TM7	0.002187±0.0007248	0.0009700±0.0003831	0.1549
Deferribacteres	0.001541±0.0005151	0.0004616±0.0002204	0.0700
Tenericutes	00.000594389	0	

Table S8 The composition of the flora community at the level of class in cecum contents (T-test)

Composition	Control	5-Fu	P value
Verrucomicrobiae	0.3007±0.06402	0.4991±0.03395	0.0135 *
Bacteroidia	0.3007±0.06402	0.3162±0.03796	0.8374
Clostridia	0.2828±0.03895	0.1471±0.02481	0.0088 **
Eosilonproteobacteia	0.02848±0.01498	0.003005±0.001033	0.1070
Grammaproteobacteria	0.02528±0.01890	0.006593±0.003346	0.3430
Alphaproteobacteria	0.006536±0.001600	0.00005132±0.00001819	0.0007***
Deltaproteobacteria	0.01481±0.002172	0.01946±0.005544	0.4444
Erysipelotrichia	0.003592±0.0008929	0.003617±0.0009385	0.9849
Bacilli	0.001685±0.0004087	0.002254±0.0007671	0.5207
Deferribacteres	0.00154063		
Unclassified	0.005586±0.001437	0.002113±0.0004704	0.0338 *
Nollocutes	0.000594389		
Actinobacteri			

Table S9 The composition of the flora community at the level of order in cecum contents (T-test)

Composition	Control	5-Fu	P value
Verrucomicrobiales	0.3007±0.06402	0.4991±0.03395	0.0135 *
Bacteroidales	0.3284±0.07683	0.3162±0.03796	0.8877
Clostridiales	0.2828±0.03895	0.1471±0.02481	0.0088 **
Campylobacterales	0.02848±0.01498	0.003005±0.001033	0.1070
Enterobacteriales	0.02507±0.01889	0.006409±0.003345	0.3435
Rhodospirillales	0.02507±0.01889	0.00005132±0.00001819	0.2019
Desulfovibrionales	0.01481±0.002172	0.01946±0.005544	0.4444
Erysipelotrichales	0.003592±0.0008929	0.003617±0.0009385	0.9849
Lactobacillales	0.003592±0.0008929	0.002254±0.0007671	0.2708
Deferribacterales	0.001541±0.0005151	0.0004616±0.0002204	0.0700
Unclassified	0.005915±0.001555	0.001026±0.0003808	0.0068 **
Coriobacterales	0.0002655±0.00005982	0.001087±0.0003288	0.0243 *
Pseudomonadales	0.0002146±0.00004695	0.0001840±0.00003676	0.6135

Table S10 The composition of the flora community at the level of family in cecum contents (T-test)

Composition	Control	5-Fu	P value
Verrucomicrobiaceae	0.2314±0.05465	0.4991±0.03395	0.0005 ***
Provotellaceae	0.04876±0.01510	0.01844±0.008162	0.0944
Unclassifed	0.005915±0.001555	0.2317±0.05744	0.0010 ***
Lachnospiraceae	0.1813±0.03122	0.06647±0.01143	0.0033 **
Helicobacteraceae	0.02848±0.01498	0.003005±0.001033	0.1070
Ruminococcaceae	0.09544±0.01282	0.05507±0.008728	0.0180 *
Bacteroidaceae	0.02718±0.007717	0.02695±0.004670	0.9796
Rikenellaceae	0.01653±0.004766	0.01273±0.003335	0.5220
Enterobacteriaceae	0.03553±0.02653	0.001483±0.0004226	0.1910
Porphyromonadaceae	0.01925±0.005374	0.008768±0.003003	0.1060
Rhodospirillaceae	0.006536±0.00600	0.00005132±0.00001819	0.0007 ***
Peptococcaceae	0.005148±0.001245	0.002500±0.0004356	0.0599
Desulfovibrionaceae	0.01481±0.002172	0.01946±0.005544	0.4444
Erysipelotrichaceae	0.003592±0.0008929	0.003617±0.0009385	0.9849
Lactobacillaceae	0.001685±0.0004087	0.002254±0.0007671	0.5207
Coriobacteriaceae	0.0002655±0.00005982	0.001087±0.0003288	0.0243 *
Defluviitaleaceae	0.001467±0.0004206	0.0002832±0.00008852	0.0130 *
Deferribacteraceae	0.001541±0.0005151	0.0004616±0.0002204	0.0700
Moraxellaceae	0.0002146±0.00004695	0.0001840±0.00003676	0.6135
Propionibacteriaceae			

Table S11 The composition of the flora community at the level of genus in cecum contents (T-test)

Composition	Control	5-Fu	P value
Akkermansia	0.3007±0.06402	0.4991±0.03395	0.0135 *
Alloprevotella	0.03761±0.01341	0.01793±0.007925	0.2223
Unclassifed	0.4034±0.05140	0.3519±0.02861	0.3931
Blautia	0.1039±0.02636	0.03301±0.01495	0.0311 *
Helicobacter	0.02848±0.01498	0.003005±0.001033	0.1070
Anaerotruncus	0.01379±0.002957	0.007140±0.001857	0.0728
Bacteroides	0.02718±0.007717	0.02695±0.004670	0.9796
Odoribacter	0.0029127		
Parabacteroides	0.01633±0.004065	0.008768±0.003003	0.1518
Ruminococcus	0.002033±0.0008483	0.003741±0.001548	0.3461
Alistipes	0.006536±0.001600	0.00005132±0.00001819	0.0007 ***
Desulfovibrio	0.01250±0.003088	0.009522±0.002762	0.4819
Oscillibacter	0.008491±0.0009494	0.01775±0.005683	0.1256
Rikenella	0.01271±0.004157	0.004426±0.0009961	0.0684
Bilophila	0.0009718±0.0004100	0.003209±0.0009055	0.0371 *
Coprococcus	0.006316±0.001997	0.001716±0.0004276	0.0370 *
Roseburia	0.007069±0.001690	0.003934±0.001230	0.1511*
Lactobacillus	0.001019±0.0003167	0.002077±0.0005648	0.1130
Enterorhabdus	0.001685±0.0004087	0.002254±0.0007671	0.5207
Candidatus Saccharimonas	0.0002655±0.00005982	0.001087±0.0003288	0.0243 *
Candidatus Arthromitus	0.002187±0.0007248	0.0009700±0.0003831	0.1549
Marvinbryantia	0.000399286		
Mucispirillum	0.0001978±0.00004584	0.0007044±0.0002342	0.0479 *
Turicibacter	0.001541±0.0005151	0.0004616±0.0002204	0.0700
Acinetobacter	0.0007593±0.0002504	0.0004597±0.0002337	0.3933
Propionnibacterium	0.0002146±0.00004695	0.0001840±0.00003676	0.6135
Thalassospira			

Table S12 The composition of the flora community at the level of species in cecum contents (T-test)

Composition	Control	5-Fu	P value
Unclassified	0.7847±0.03792	0.7725±0.03184	0.8078
Uncultured bacyeroidales bacterium	0.09597±0.03631	0.1617±0.02930	0.1762
Helicobacter ganmani	0.02848±0.01498	0.003005±0.001033	0.1070
Anaerotruncus sp. G3	0.01256±0.002904	0.005846±0.001412	0.0523
Gut metagenome	0.021095		
Bacteroides vulgatus(ATCC 8428)	0.002781±0.001524	0.01700±0.004454	0.0073 **
Eubacterium coprostanoligenes	0.00326508		
Firmicutes bacterium ASF500	0.01333±0.003497	0.004081±0.0007758	0.0188 *
Parabacteroides goldsteinii	0.001758±0.0009900	0.006612±0.002554	0.0932
Clostridium papyrosolvens	0.004695±0.003216	0.0006296±0.0004238	0.2261
Mouse gut metagenome	0.004054±0.001139	0.007068±0.003249	0.3930
Bacteroides acidifaciens	0.004621±0.001216	0.005861±0.001077	0.4549
Lachnospiraceae bacterium 610	0.005703±0.003249	0.000008447±0.000008447	0.0967
Uncultured rikenellaceae bacterium	0.0001016±0.00005553	0.001995±0.0004610	0.0007 ***
Bacteroids uniformis	0.0001741±0.00007605	0.002450±0.0006886	0.0041 **
Desulfovibrio sp. ABHU1SB	0.0009436±0.0005257	0.002500±0.001007	0.1874
Unidentified rumen bacterium RFN71	0.001332±0.0005893	0.001433±0.0002636	0.8775
Clostridium sp. ABHU1SB	0.0004405±0.00008135	0.0004785±0.0002046	0.8651
Bacteroides bacterium ph8	0.0001064±0.00006598	0.0002876±0.00007040	0.0768
Lachnospiraceae bacterium 10-1	0.0009771±0.0005363	0.0001268±0.00004718	0.1316
Dorea sp. 5-2	0.001606±0.0003919	0.001874±0.0004092	0.6413
Clostridium sp. Culture-41	0.002975±0.0008249	0.001083±0.0005509	0.0725
Clostridiales bacterium CIEAF 020	0.00287277		
Clostridiales bacterium CIEAF 012	0.0001978±0.00004584	0.0007044±0.0002342	0.0479 *
Unclutured clostridiales bacterium		0.000808674	
Unclutured rumen bacterium	0.001336±0.0008452	0.00005132±0.00001819	0.1461
Bacteroides stercoris(ATCC 43183)	0.00004030±0.00001657	0.00002747±0.00001878	0.6146
Clostridium sp. Culture-27	0.001982±0.001365	0.00005977±0.00005977	0.1766
Clostridium leptum	0.0004466±0.0001533	0.0004523±0.0001761	0.9809
Lactobacillus murinus	0.001417±0.0003773	0.0008577±0.0002453	0.2295
Clostridium sp. ATCC 29733		0.000525516	

Fig.S1

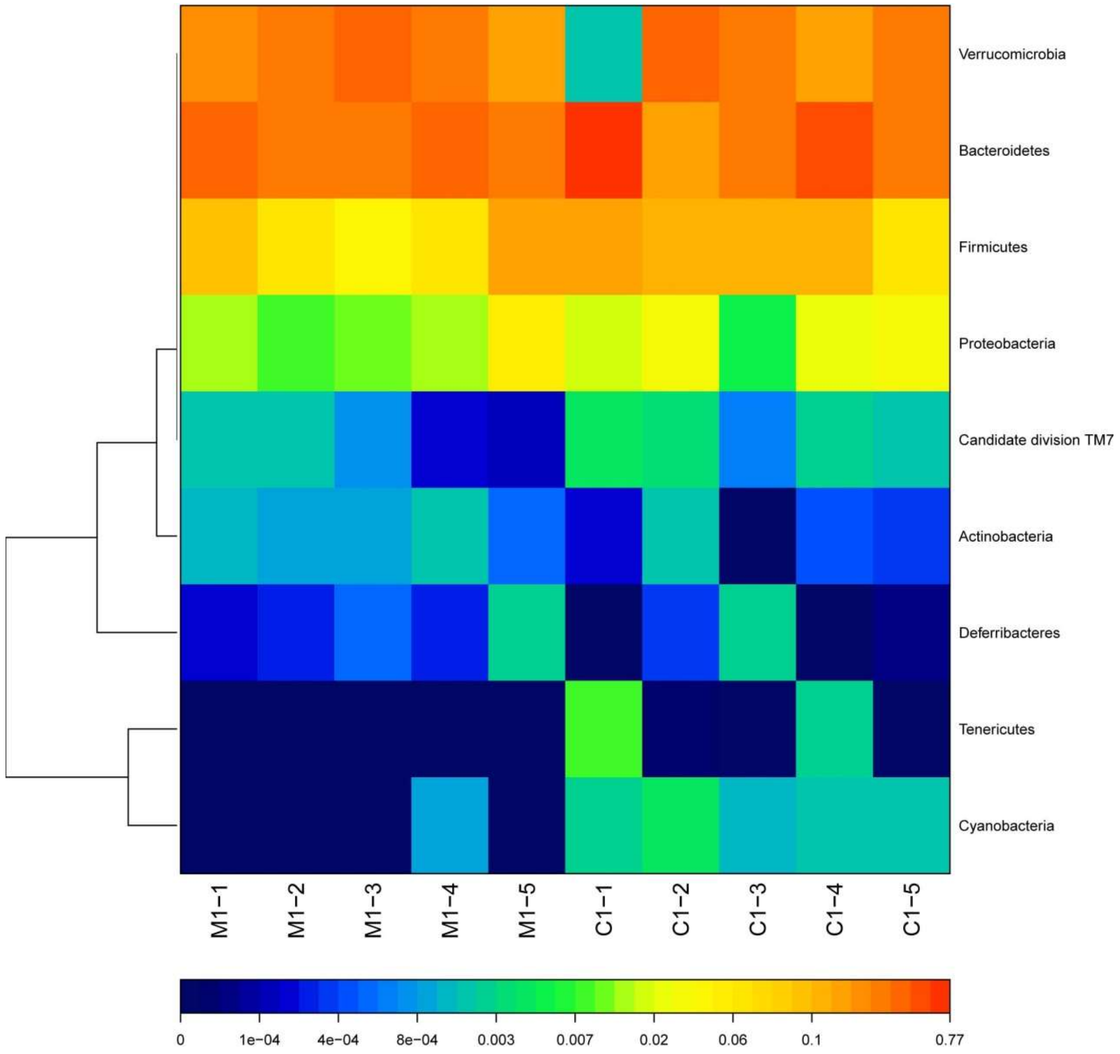

The heatmap of relative abundance of gut microbiota at phylum level in feces of mice
M: 5-Fu treated mice; C: normal mice

Fig.S2

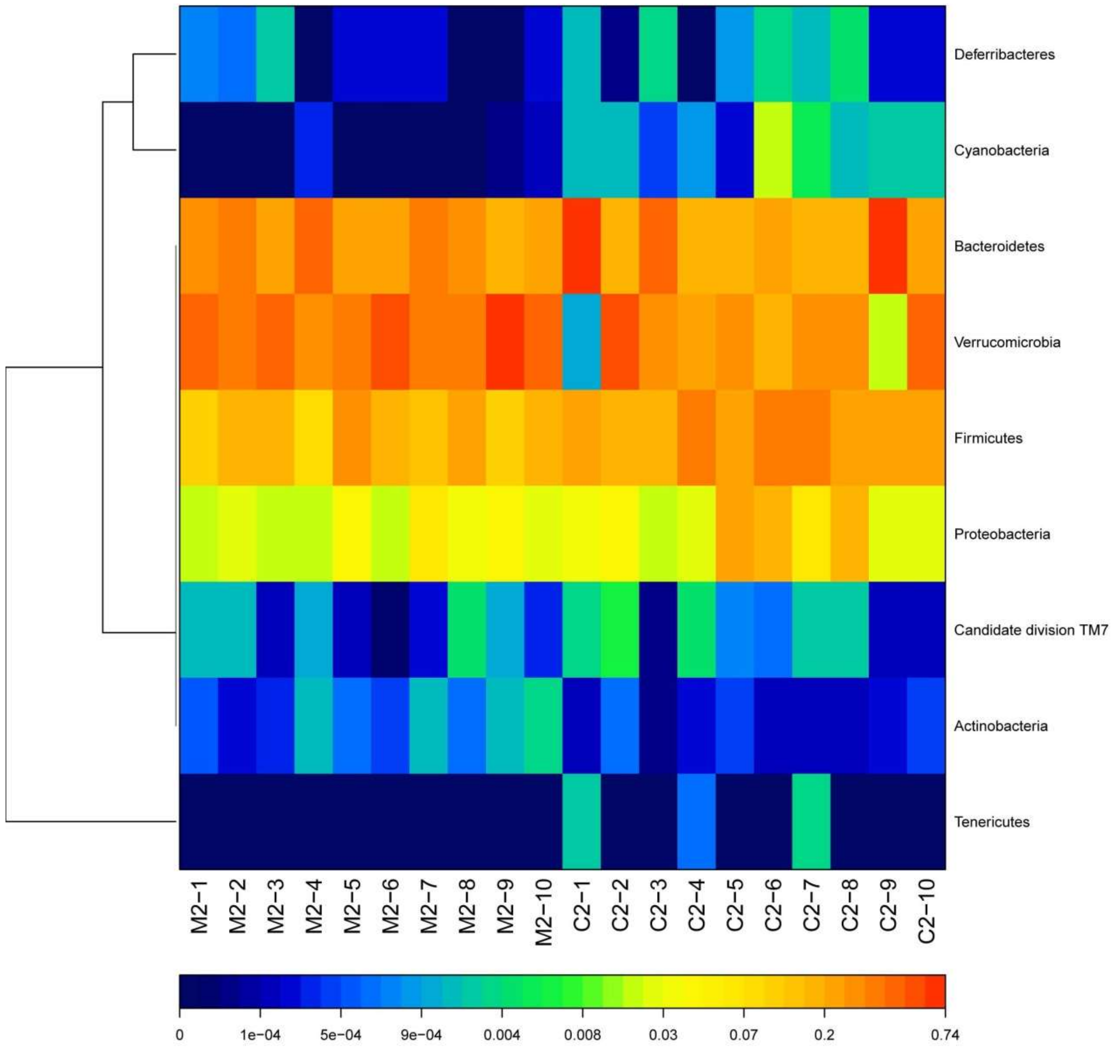

The heatmap of relative abundance of gut microbiota at phylum level in cecum contents of mice
M: 5-Fu treated mice; C: normal mice

Fig.S3

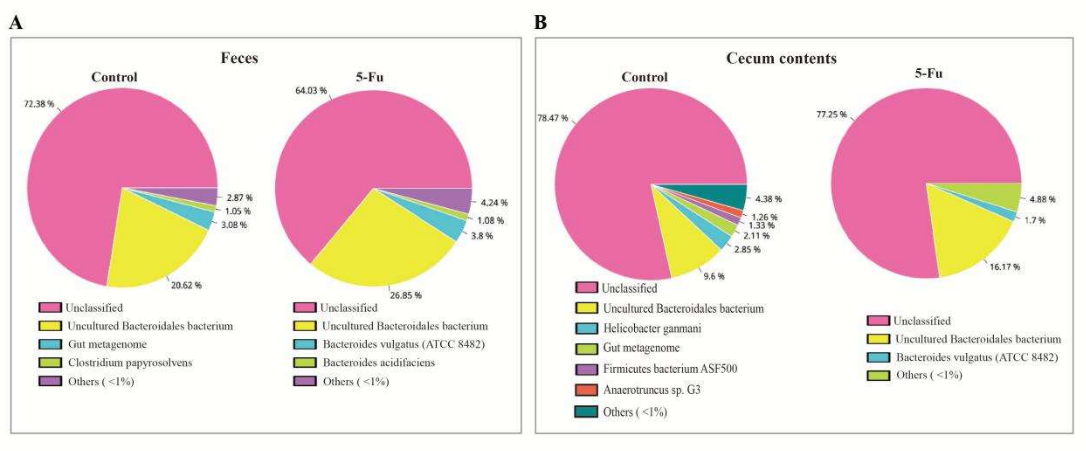

The pie charts depicting the mean relative abundances for feces and cecum contents at species level

Fig.S4

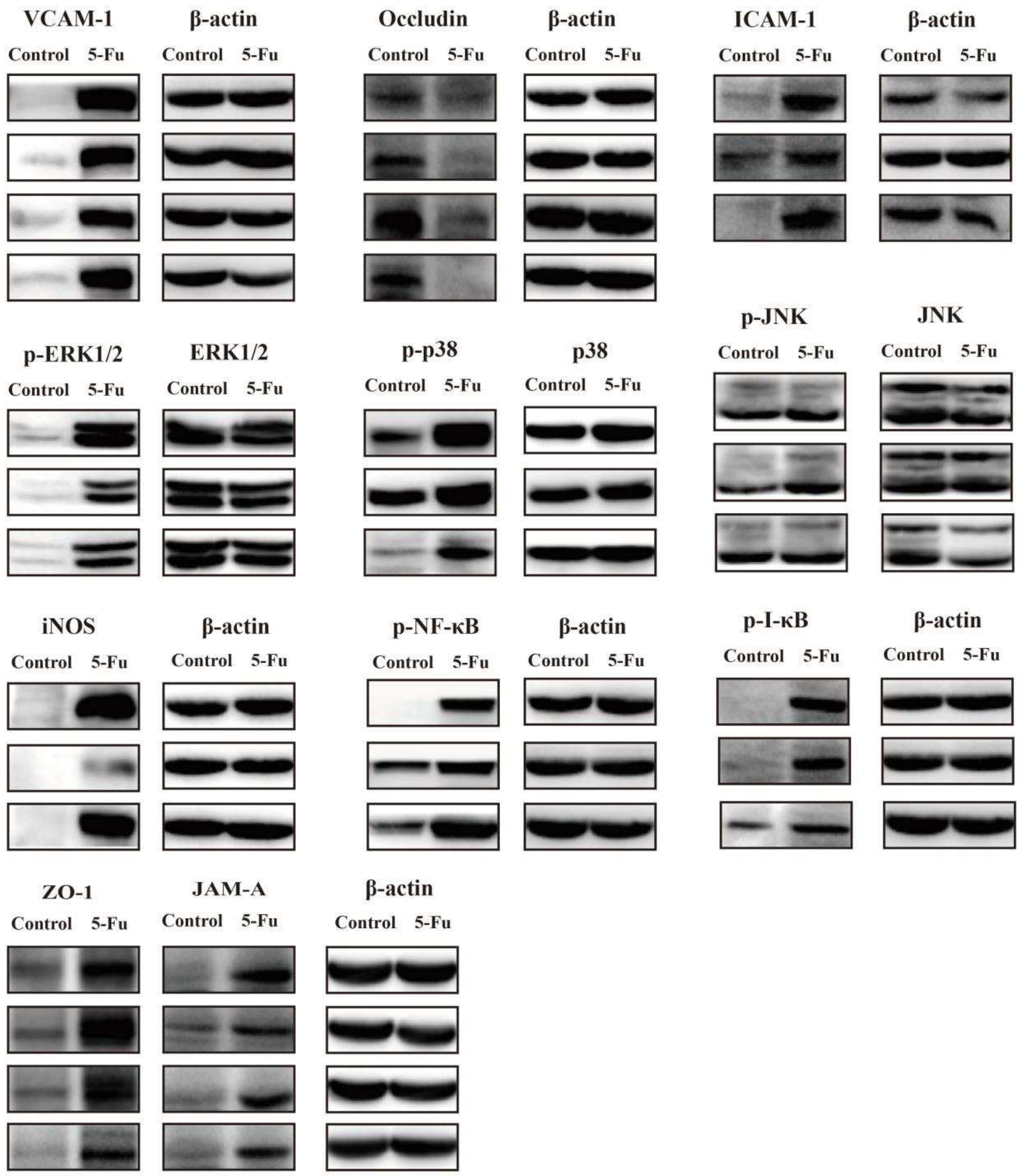

All the WB images for all the mice used in present study
